# Proteomic profiling across breast cancer cell lines and models

**DOI:** 10.1038/s41597-023-02355-0

**Published:** 2023-08-04

**Authors:** Marian Kalocsay, Matthew J. Berberich, Robert A. Everley, Maulik K. Nariya, Mirra Chung, Benjamin Gaudio, Chiara Victor, Gary A. Bradshaw, Robyn J. Eisert, Marc Hafner, Peter K. Sorger, Caitlin E. Mills, Kartik Subramanian

**Affiliations:** 1 Laboratory of Systems Pharmacology, Program in Therapeutic Science, Harvard Medical School, Boston, MA 02115 USA; 2 Present Address: Department of Experimental Radiation Oncology, The University of Texas MD Anderson Cancer Center, Houston, TX 77030 USA; 3 Present Address: IGBMC, Strasbourg, Grand Est France; 4 Present Address: Department of Oncology Bioinformatics, Genentech, Inc., South San Francisco, CA 94080 USA; 5 Present Address: Bristol Myers Squibb, Cambridge, MA 02142 USA

**Keywords:** Breast cancer, Proteomic analysis

## Abstract

We performed quantitative proteomics on 60 human-derived breast cancer cell line models to a depth of ~13,000 proteins. The resulting high-throughput datasets were assessed for quality and reproducibility. We used the datasets to identify and characterize the subtypes of breast cancer and showed that they conform to known transcriptional subtypes, revealing that molecular subtypes are preserved even in under-sampled protein feature sets. All datasets are freely available as public resources on the LINCS portal. We anticipate that these datasets, either in isolation or in combination with complimentary measurements such as genomics, transcriptomics and phosphoproteomics, can be mined for the purpose of predicting drug response, informing cell line specific context in models of signalling pathways, and identifying markers of sensitivity or resistance to therapeutics.

## Background & Summary

Targeted therapy relies on the identification of actionable changes in signal transduction, proliferation or cell death pathways that are drivers of transformed states. In some cases, these changes are associated with a recurrent mutation or overexpression of an oncogene. In other cases, the causes of differences in drug sensitivity are less well understood. Some breast cancer subtypes are particularly responsive to targeted therapy owing to high expression of one or more of the estrogen (ER), progesterone (PR), or HER2 receptors. Moreover, the presence or absence of these receptors, which is most commonly measured by immunohistochemistry (IHC), defines clinical breast cancer subtype and mode of first line therapy (expression of ER and/or PR defines the hormone receptor (HR) positive subtype and over-expression of HER2/ERBB2 defines the HER2 positive subtype). The third breast cancer subtype, triple negative breast cancer (TNBC), lacks high levels of ER, PR and HER2 expression, is genetically heterogeneous and is the least effectively treated^1^. It is therefore of considerable interest to identify recurrent changes in TNBCs that might be targeted to treat disease. Breast cancers can also be classified into molecular subtypes based on gene expression signatures. These include the luminal A/B and basal designations that generally encompass HR positive and TNBC disease respectively, with HER2 enriched cancers comprising a separate molecular subtype^2–4^.

Multiple studies have been performed in which panels of TNBC cell lines are subjected to transcript profiling to identify differences among them^5,6^. However, transcript levels do not necessarily correlate with the abundance of proteins, which are the ultimate targets of small molecule and antibody therapies^7^. Moreover, in some tumor types, the effects of copy number alterations extend to mRNA abundance without necessarily propagating to changes in protein abundance^8^. It is therefore valuable to measure the levels of proteins across panels of cell lines to identify changes in cell state. Of particular interest are changes that might individually or in combination determine sensitivity to new or existing drugs for the treatment of breast cancer. It has been shown that computational models of drug sensitivity that are trained using protein expression data can complement or even outperform models trained on transcript or genomic data alone^9–11^. Thus, a standardized dataset on protein expression levels in breast cancer cell lines of all three clinical subtypes is expected to constitute a valuable resource for drug discovery and development of predictive biomarkers.

In this data descriptor we describe systematic profiling of 60 widely used breast cancer cell lines using Tandem Mass Tag (TMT) liquid chromatography mass spectrometry (LC/MS). TMT LC/MS is a method for labelling multiple samples (up to 11 in the current work) with mass tags and then analyzing them in parallel on the mass spectrometer, thereby enabling direct comparison of protein levels. In this data descriptor we provide a technical summary of the TMT-based mass spectrometry approach and the resulting data. Quality metrics used to asses both technical and biological validity are explained and we highlight the use of bridge samples to enable normalization and comparison of samples collected in the face of technical and methodological advances. We discuss how the resulting data can be leveraged to characterize preclinical (cell line) models of breast cancer, generate testable hypotheses of resistance to therapy and discover novel biological insight.

## Methods

### Culture conditions

All cell lines used in this study were of human female origin and derived from breast cancers except the 184A1, 184B5, MCF 10A, MCF12A and HME1 cell lines which were derived from non-malignant human breast epithelia. Cell lines were maintained, free of mycoplasma, in their recommended growth conditions (see Supplementary Table 1), and were identity-validated by STR profiling^12^.

### Mass spectrometry

**Fig. 1:**
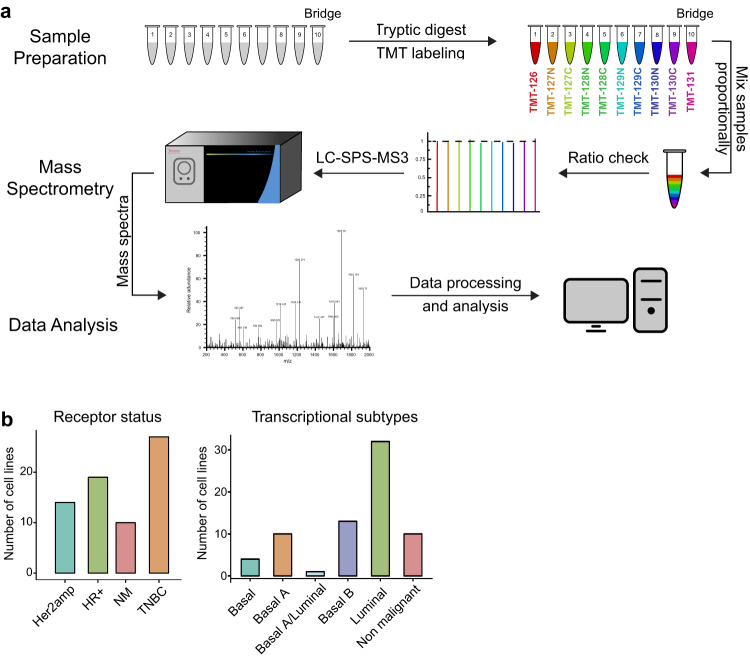
Experimental overview (**a**) Mass spectrometry workflow. Cell pellets were lysed and either 65 or 150 ug of protein of each sample was labelled using a TMT Mass Tag Labelling Kit (see methods for details of differences between sets). TMT labelled samples were pooled into a single multiplexed sample and a ratio check was performed to ensure that an equal amount of each TMT label was included. The samples were injected into an Orbitrap Fusion Lumos Tribrid mass spectrometer, and TMT quantification was performed in the Orbitrap using SPS-MS3. Assignment of MS/MS spectra was performed using Sequest. (**b**) Classification of breast cancer cell line samples included in the study based on molecular subtype (left panel) or receptor status (right panel).

A schematic description of our mass spectrometry workflow is shown in Fig. 1a. Data were collected in 8 separate mass spectrometry sets. Because data collection spanned many months and instrumentation and protocols improved over this period, methods differed between batches (batch 1 includes sets 1–4 and batch 2 includes sets 5–8) as described below.

#### Sample collection

Cells grown in their recommended growth medium to ~60% confluence were rinsed twice with phosphate-buffered saline (PBS) and then gently scraped from 15 cm dishes in PBS supplemented with protease and phosphatase inhibitors (Halt^TM^ Protease and Phosphatase Inhibitor Single-Use Cocktail, EDTA Free, ThermoFisher, Catalog Number 78441) followed by centrifugation at 300 g for 5 minutes at 4 °C. The supernatant was discarded, and pellets were snap frozen in liquid nitrogen and stored at −80 °C.

#### Protein solubilisation and digestion

Cell pellets were solubilized in lysis buffer (2% SDS, 150 mM NaCl, 50 mM Tris pH 7.4) supplemented with Halt™ Protease and Phosphatase Inhibitor Single-Use Cocktail, EDTA Free (ThermoFisher, Catalog Number 78443) with a hand-held tissue homogenizer. Disulfide reduction was performed by adding dithiothreitol (DTT) to a final concentration of 5 mM and heating to 37 °C for 1 hour, followed by alkylation of cysteine residues with iodoacetamide at a final concentration of 15 mM and incubation at room temperature in the dark for 30 minutes. Protein concentration was determined using a Micro BCA™ Protein Assay Kit (ThermoFisher, Catalog Number 23235) following the manufacturer’s protocol. Detergent was removed by methanol/chloroform protein precipitation as follows: ice cold methanol (3 parts lysis buffer volume), chloroform (2 parts lysis buffer volume) and water (2.5 parts lysis buffer volume) were added sequentially with vortexing after each addition followed by centrifugation at 4000 × g for 10 min. The top layer was aspirated while taking care not to disrupt the interface. Ice cold methanol (3 parts lysis buffer) was added, the samples were vortexed, centrifuged (4000 × g, 10 min.), and the supernatant aspirated leaving behind the protein pellet; this methanol wash procedure was repeated a total of three times^13^. Precipitates were solubilized in freshly prepared 8 M urea in 200 mM EPPS, pH 8.5. Following a 10 min incubation at 37 °C, the urea concentration was reduced by dilution with 200 mM EPPS, pH 8.5 to 4 M (sets 1–4) or 2 M (sets 5–8) final urea concentration and digestion was then performed by overnight incubation at room temperature in the presence of Lys-C protease (Wako, Catalog Number 129-02541) at an enzyme-to-substrate ratio of 1:75. Following further dilution of the sample with 200 mM EPPS to a final urea concentration of 1.6 M (sets 1–4) or 0.5 M (sets 5–8), digestion was continued by incubation of the sample at 37 °C for 6 hours with trypsin (Promega, Catalog Number V5113) at an enzyme to substrate ratio of 1:75. Aliquots corresponding to 65 µg per sample (sets 1–4) or 150 µg per sample (sets 5–8) were withdrawn for TMT labelling.

#### Digest check

Aliquots equivalent to 5–10 µg from two samples were pooled, desalted and peptides purified by reverse phase chromatography on stage tips^14^ (described below). The peptides were then dried and resuspended in 3% acetonitrile, 5% formic acid (FA) to a final concentration of ~2 µg/µl. The missed cleavage rate was measured by LC-MS/MS to evaluate the quality of the digest; a result under 15% of potential cleavage sites remaining uncleaved was deemed sufficient to proceed with labelling.

#### TMT labelling, ratio check and hplc fractionation

Equal amounts of protein were removed from each sample and labelled using a TMT10plex or TMT11plex Mass Tag Labelling Kit (ThermoFisher, Catalog Number A34808) (see Supplementary Table 2 for sample labeling metadata for each Set and Batch). TMT labelling efficiency was measured by LC-MS3 analysis after combining equal volumes (equivalent to ~ 1 µg each) from each sample. At this stage a ratio check was performed in which the total peptide intensities from each sample were compared for equivalence. Equal amounts of labelled peptide from each sample (as judged from ratio check data) were then combined for subsequent fractionation in a single HPLC run (see below); each set involved a total amount of approximately 600 µg protein. Quenching of TMT labelling reactions was performed by adding hydroxylamine to a final concentration of 0.5% (v/v) and incubating samples for 15 minutes at room temperature. Formic acid (FA) was added to a final volume of 2% (v/v) to lower the pH below 3.0 and samples were combined and de-salted using a SepPak tC18 Vac RC Cartridge (50 mg, Waters, Catalog Number WAT054960). HPLC fractionation was performed over a period of 75 minutes using an Agilent 1200 Series instrument with a flow rate of 600 µl/minute. Peptides were collected in a 96-well plate over a 65 min-gradient of 13–44%B with Buffer A comprising 5% acetonitrile, 10 mM ammonium bicarbonate, pH 8 and Buffer B comprising 90% acetonitrile, 10 mM ammonium bicarbonate, pH 8. Fractions were pooled to generate a total of 24 aliquots, followed by sample clean-up using the Stage Tip protocol with C18 Empore™ Extraction Disks (Fisher Scientific, Catalog Number 14-386-2). The matrix was primed with methanol and equilibrated with 70% acetonitrile, 1% FA followed by washing twice with 1% FA. Samples were loaded in 1% FA, followed again by two 1% FA washes, and finally peptides were eluted using 70% acetonitrile, 1% FA. Samples were dried before resuspension in MS Loading Buffer (3% acetonitrile, 5% FA).

#### LC-MS

The first half of the dataset (sets 1–4) was recorded after peptide separation on 100 µm columns packed with 1.8 µm C18 beads with a pore size of 12 nm (Sepax Technologies Inc.). The second half of the data (sets 5–8) was obtained after peptide separation on 75 µm columns packed with 2.6 µm Accucore beads (Thermo Fisher Scientific). Peptides were injected onto 30–40 cm, 100 and 75 µm (internal diameter) columns, respectively, and separated using an EASY-nLC 1200 HPLC (ThermoFisher Scientific). The flow rate was 450 nl/min for the 100 µm columns and 300 nl/min for the 75 µm columns with a gradient of 6–28%B over 170 minutes with Buffer A comprising 3% acetonitrile, 0.4% FA and Buffer B comprising 100% acetonitrile, 0.4% FA for the 100 µm columns and 5–35%B over 240 minutes with Buffer A comprising 0.125% FA and Buffer B comprising 95% acetonitrile, 0.125% FA for the 75 µm columns. The columns were heated to 60 °C using a column heater (constructed in-house). Samples from the HPLC were injected into an Orbitrap Fusion Lumos Tribrid MS (ThermoFisher, Catalog Number FSN02-10000) using a multi-notch MS3 method^15,16^. MS scans were performed in the Orbitrap over a scan range of 400–1400 m/z with dynamic exclusion. Rapid rate (sets 1–4) and Turbo rate (sets 5–8) scans were performed in the Ion Trap with a collision energy of 35% and maximum injection times of 120 ms (sets 1–4) and 200 ms, (sets 5–8) respectively. TMT quantification was performed using SPS-MS3 in the Orbitrap with a scan range of 100–1000 m/z and an HCD collision energy of 55%. Orbitrap resolution was 50,000 (dimensionless units) with maximum injection times of 120 ms (sets 1–4) and 450 ms (sets 5–8), respectively. MS isolation windows were varied depending on the charge state. Additional reruns of fractions were performed for the later sets to achieve increased depth. Details are provided in Supplementary Table 3 including a mapping of deposited.raw file names to individual sets.

### Data analysis

Mass spectrometric data (Thermo “.RAW” files) were converted to mzXML format, to correct monoisotopic m/z measurements, and to perform a post-search calibration. Peptide spectrum matches were assigned with SEQUEST (v.28 (rev. 12), (c) 1998–2007 Molecular Biotechnology, Univ. of Washington, J.Eng/S.Morgan/J.Yates licensed to Thermo Fisher Scientific Inc.) based software. The quality of peptide identifications by SEQUEST was determined with a target-decoy approach, where each peptide spectra was searched against a composite database of size-sorted forward and reverse protein sequences of the human proteome (Uniprot 02/2014) that also contained common contaminant proteins. For each peptide, identification scores (X_corr_, ΔCn, and precursor mass error) were computed by SEQUEST for target and decoy hits. Linear discriminant analysis that combines the 3 SEQUEST identification score parameters into an optimal discriminant score was performed^17,18^. For each set, the false discovery rate (FDR) was computed as twice the number of reverse peptides identified divided by the total number of peptide identifications above any given discriminant score threshold^19^. Peptides were filtered to achieve an FDR <1%. During peptide assignment for all data, oxidized methionine (+15.9949 Da) was searched dynamically. All peptide searches considered TMT modification (+229.1629 Da) on N-termini and lysine residues as static modifications. For each set, the FDR for protein identification was set to <1%^17^, and shared peptides were then collapsed into proteins using rules of parsimony, i.e if a peptide could be mapped to multiple proteins, it was assigned only to the largest protein^17^. Each peptide was assigned only to one protein. Peptides with a total TMT value of >200 and an isolation specificity of >0.7 were included in the final dataset.

## Data Records

MS proteomics Level 0 Data on peptides have been deposited to the ProteomeXchange Consortium via the PRIDE^20^ partner repository with the dataset identifier PXD026581: https://identifiers.org/pride.project:PXD026581^21^.

Proteome datasets are available on Figshare: 10.6084/m9.figshare.c.6443633.v2^22^ and Synapse: 10.7303/syn32672593; these data include Level 1 data on peptide intensities (Synapse ID: syn32672684), Level 2 data on peptide intensities normalized within and across sets (Synapse ID: syn32672825) and Level 3 data (Synapse ID: syn32672858) in which protein level data is derived from the mean peptide intensities^23^.

## Technical Validation

### Mass spectrometry instrument quality control

Quality control checks for mass spectrometry were incorporated at multiple points in the workflow. To test for efficient digestion of samples, defined as <15% of potential proteolysis sites uncleaved, a “digest check” was performed using LC-MS/MS as described in the methods section. TMT labelling efficiency aims for modification of >95% of available sites and was determined by LC-MS analysis via dynamic searches for N-terminal peptide modification by TMT. A “ratio check” was also performed using LC-MS3 to determine relative amounts of labelled peptides in each of the multiplexed samples, as described in the methods section. The purpose of the ratio check is to ensure equal amounts of peptide across all samples in a set are pooled in the sample run through the mass spectrometer.

### Reproducibility of results

73 samples (60 unique breast cancer cell lines and 13 technical or biological replicates) distributed across three breast cancer clinical subtypes (Fig. 1b) were randomly divided into 8 sets. Each set had one or more bridge samples that comprised a mixed sample derived from six cell lines (HCC1806, Hs578T, MCF7, MCF 10A, MDAMB231, SKBR3). By including the same bridge sample in each MS set it was possible to compare sets to each other (see below). A total of five biological replicates of the MCF 10A cell line were also present in the eight sets as a further measure of data reproducibility.

**Fig. 2:**
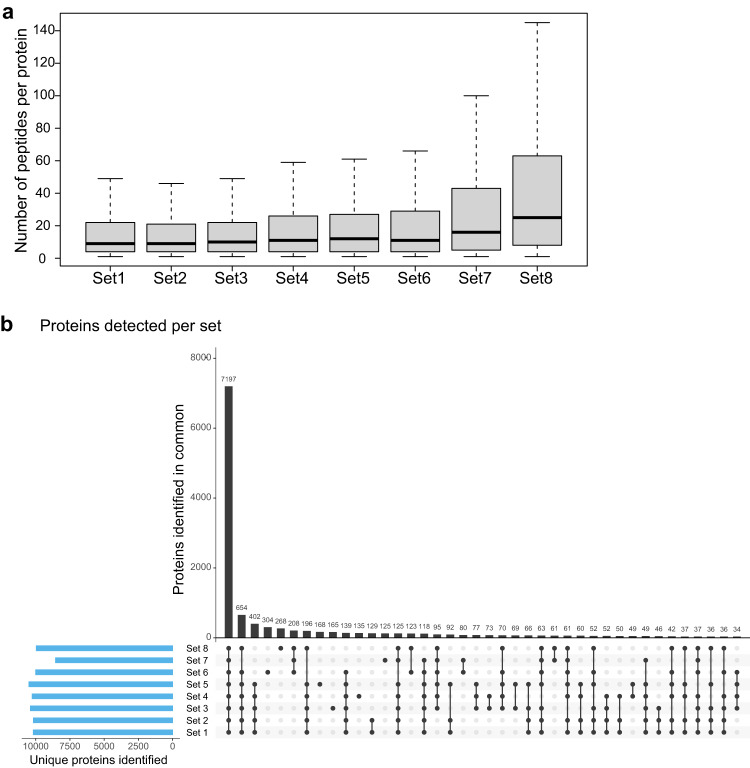
Summary of peptides and proteins detected (**a**) Box and whisker plot showing the distribution of peptides detected per protein for each set. The box represents the first quartile, median value, and third quartile and the error bars represent the maximum and minimum. (**b**) UpSet plot to illustrate the depth in protein coverage for each of the eight sets and the number of overlapping proteins across them.

Principal component analysis (PCA) was performed prior to data normalization and revealed a significant degree of clustering by batch (sets 1–4 versus sets 5–8). This was true despite the high overlap in proteins detected. We could identify two reasons for this batch effect: advances in instrumentation and analytical methods meant that later batches exhibited better signal to noise as assessed by an increase in mean intensity per protein. Samples in the second batch had a greater number of quantifiable total peptides per protein (Fig. 2a). This difference between the two batches may be due to the longer gradients used for peptide separation prior to MS analysis in the second batch (170 min for sets 1–4 versus 240 min for sets 5–8) and the fact that we re-shot some fractions (6 in set 6; 16 in set 7; and all 24 in set 8) in the second batch. Out of 19,000–22,000 known human proteins^24^, we measured a total of ~13,000 unique proteins in our dataset. 7197 proteins were detected in all sets while the remaining proteins were observed to varying degrees in different sets (Fig. 2b). In shotgun proteomics, there is variation in the number of proteins detected in each MS set due to under-sampling and differences from one sample to the next can therefore reflect both real biological variation and under-sampling. To correct for the differences in the number of quantifiable peptides per protein between batches, the peptide intensities in each sample of a set were normalized to the bridge sample for that set so that the summed peptide intensity scores across all samples were equivalent (within set normalization of samples Eq. 1). Next, all peptide intensities were normalized to the data from the bridge sample of a reference set (set 4 in this study) to allow for comparisons across sets and batches (Eq. 2). To account for variability in the number of peptides reported for a given protein across sets (Fig. 2b), intensities for each protein in a set were calculated by averaging across all peptides reported for that protein in any given sample of a set (Eq. 3). Finally, for each set, the normalized peptide and protein intensities computed in Eqs. 2 and 3 respectively were scaled to a range from 0 to 100 (Eq. 4).

1. *Within* set normalization at the peptide level: Each peptide, *x*_*i*_ in sample *j* and set *k* was normalized by multiplying its intensity by the ratio of the summed intensities of sample *j* and bridge sample (*j*^*b*^) in set *k*
  1 $${\ddot{x}}_{ijk}={\dot{x}}_{ijk}\ast \frac{{\sum }_{i}^{n}{\dot{x}}_{{j}^{b}k}}{{\sum }_{i}^{n}{\dot{x}}_{jk}}$$
2. *Across* set normalization at the peptide level: Each within-set normalized peptide, ${\ddot{x}}_{i}$ in sample *j* and set *k* was normalized by multiplying its intensity by the ratio of the summed intensities of bridge sample in the reference set (*j*^*b*^ *k*^*r*^) and bridge sample in set *k* (*j*^*b*^ *k*)
  2 $${\mathop{x}\limits^{...}}_{ijk}={\ddot{x}}_{ijk}\ast \frac{{\sum }_{i}^{n}{\dot{x}}_{{j}^{b}{k}^{r}}}{{\sum }_{i}^{n}{\dot{x}}_{{j}^{b}k}}$$
3. Derivation of protein level data: Quantity of any protein, *y*_*l*_ in sample *j* and set *k* was calculated by summing across all normalized peptide intensities, reported for protein *y*_*l*_ in sample *j* and set *k* and dividing by the number of peptides *n*_*lk*_ reported for protein *y*_*l*_ in set *k*
  3 $${y}_{ljk}=\frac{{\sum }_{i}^{n}{\mathop{x}\limits^{...}}_{ijk}}{{n}_{lk}}$$
4. Scaling of peptide and protein intensity data: Quantity of any peptide or protein in each set *k* was scaled to a value between 0 to 100 by dividing the normalized intensity of each peptide or protein *x*_*ijk*_ (calculated in Eqs. 2, 3 respectively) by the summed normalized intensities of protein/peptide *x*_*i*_ across all samples *j* in set *k*:

4 $${\bar{x}}_{ijk}=100\ast \frac{{x}_{ijk}}{{\sum }_{j}^{n}{x}_{ijk}}$$

### Biological validation

**Fig. 3:**
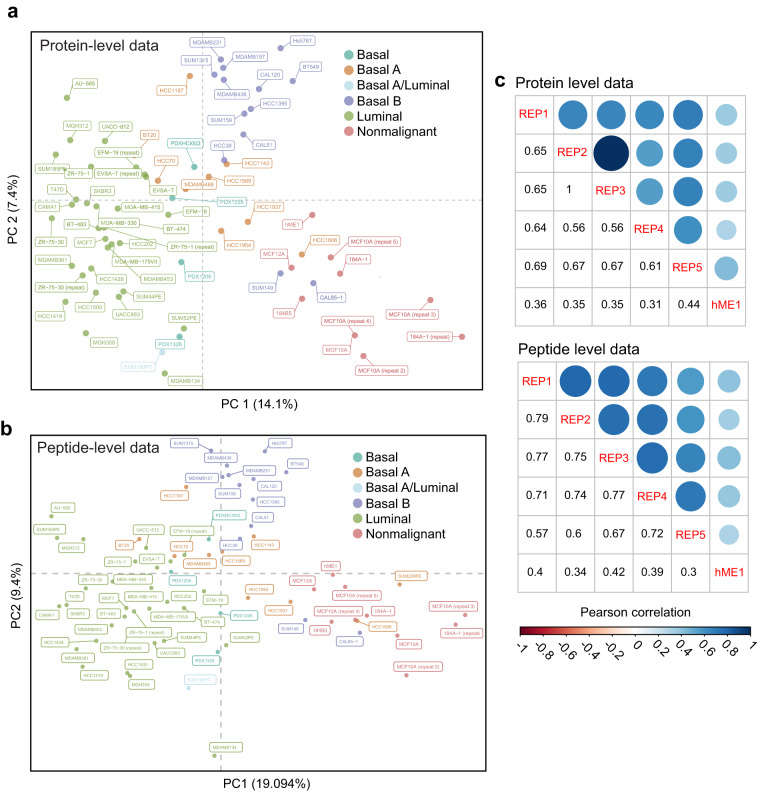
Assessment of data quality (**a**) Principal Component Analysis (PCA) of the protein-level normalized dataset corrected for set-specific differences in intensities and number of peptides quantified per protein. Cells clustered according to their known transcriptional subtypes. (**b**) Principal Component Analysis (PCA) of the peptide-level normalized dataset. Cells clustered according to their known transcriptional subtypes. (**c & d**) Correlation between normalized protein and peptide level data of technical replicates of a single cell line (the non-malignant MCF 10A cell line) across five batches. HME1 is included to show contrast with another non-malignant cell line.

After normalization, PCA-based clustering of the 74 samples at the peptide and protein levels showed that data for each cell line clustered by transcriptional subtype (Fig. 3a,b), suggesting that normalization was effective in removing batch effects and that both peptide and protein features in the data capture the intrinsic heterogeneity of the cell line panel as previously established by transcript profiling. MCF 10A replicates included in five sets covering both batches clustered together, another indication that any remaining batch effects were small (Fig. 3a,b).

## Usage Notes

### Availability of data at different levels of processing

The NIH LINCS program has defined different data levels for all data, including proteomics, that comprise: Level 0 (primary or raw;.raw files and mzXML files in the case of MS data), Level 1 (relative peptide level intensities reported for each set), Level 2 (batch normalized peptide intensities reported for each set), Level 3 (sub-threshold proteinand contaminant proteins removed, and batch normalized) and Level 4 (signatures and markers of response)^25^ (these data levels are described in detail in the accompanying overview manuscript). Level 0 data for the current study are available for download from the Pride repository (PXD026581)^21^. Level 1 (available on Synapse: syn32672684^23^) data were generated using software provided by the Gygi Laboratory at HMS and comprise peptide level estimates. The peptide intensity estimates in Level 1 were normalized using bridge samples to make cross-set and cross batch comparisons possible, followed by calculation of protein level estimates (Level 2 and 3 data available on Synapse: syn32672825 & syn32672858^23^, respectively). Level 1 datasets can also be generated by users from the Level 0 raw data using MaxQuant^26^. While Level 2 data can either be generated from Level 1 data as described using Eqs. (1–4) or with packages like FragPipe^27^.

### Comparison of peptide and protein level datasets

Across the 8 sets in our study, we identified 135,970 unique peptides that mapped to ~13,000 proteins, 7200 of which were common across all sets. However, only 25,925 peptides that mapped to 4820 proteins were found to be common across all sets. Therefore, for ~2380 proteins identified in common across the entire study, the underlying peptide or peptides used to make the quantification differed across sets. A caveat of deriving protein intensities by averaging normalized peptide intensities is that it relies on the assumption that different peptides used to quantify a protein are quantitatively similar and comparable^28^. To assess if this is a reasonable assumption to make for this dataset, we compared the correlation across the 5 non-malignant MCF 10A replicates and the non-malignant hME1 cell line model using either normalized protein or peptide level measurements found in common across all 8 sets (Fig. 3c). At the peptide level, the mean correlation coefficient between the 5 non-malignant MCF 10A replicates was 0.708 compared to a mean correlation coefficient of 0.719 at the protein level. In contrast, the mean correlation coefficient between any MCF 10A sample and the non-malignant hME1 model was 0.37 using peptide level data and 0.31 using protein level data. In addition; both peptide level and protein level data cluster by subtype in PCA space (Fig. 3a,b). Therefore, both peptide level and protein level quantification effectively discriminate between cell line models of the same subtype and retain correlation between technical replicates. We conclude that protein-level quantification from disparate peptides is a reasonable approach that allows for greater data retention (7200 vs 4820 unique proteins) and that meaningful comparisons can be made in relative protein abundance even if different peptides were detected in different sets. Both peptide-level and protein level normalized versions of the data (level 2 & 3 data) are available for download.

### Application to drug response studies

**Fig. 4:**
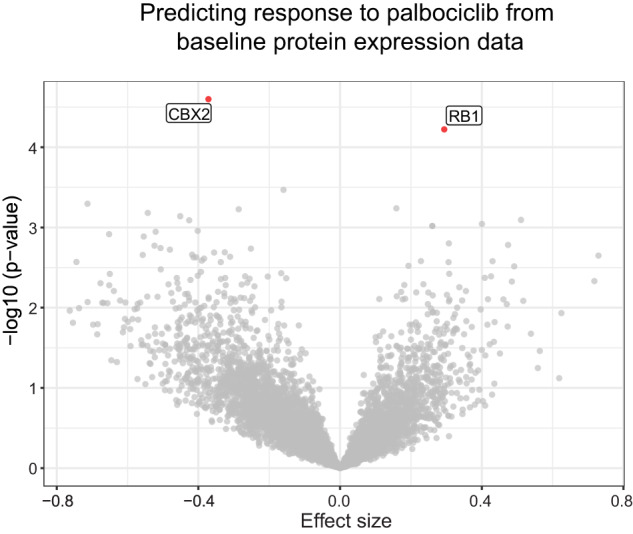
Prediction of breast cancer cell line response palbociclib treatment Volcano plot (-log10 (*p*-value) against Effect size) shows the relative strength of each of the 7197 proteins in predicting breast cancer cell line response (GR AOC) to palbociclib. Negative effect size is associated with drug resistance and positive effect size is associated with sensitivity.

Genomic and transcriptomic data have frequently been used to predict drug response and identify potential predictors or determinants of drug sensitivity^29–31^. As a first step in determining the utility of protein expression data in predicting drug response, we measured the responses of 56 breast cancer cell lines to the CDK4/6 inhibitor palbociclib (available on the LINCS database https://lincs.hms.harvard.edu/db/datasets/20343). Using the relative abundance for each of the 7197 proteins measured in all cell lines, we built univariate linear models to predict response (area over the GR curve) to palbociclib. The model included receptor status as a covariate to account for subtype specific differences in protein expression (The ‘lm’ package in R was used to encode the linear models using the formula “palbociclib GR AOC ~ protein expression + receptor status”) (Fig. 4). As expected, the abundance of RB1, a key substrate of CDK4/6 and mediator of cell cycle arrest^32^, was among the strongest predictors of response to palbociclib (*p*-value = 5.9e-06) and was positively correlated with increased sensitivity. In contrast, expression of CBX2 was correlated with resistance to palbociclib (*p*-value = 2.5e-06). Overexpression of CBX2, a protein involved in DNA damage repair and chromatin homeostasis^33,34^, has been associated with upregulation of genes involved in cell cycle progression and worse 5-year survival in breast cancer patients^35^. The association of CBX2 expression with resistance to palbociclib in cell lines provides a rationale for considering it as a potential biomarker in humans and a possible therapeutic target to overcome resistance to CDK4/6 inhibitors. This preliminary analysis suggests that baseline protein expression in untreated cell line models can be used to generate testable hypotheses about factors that influence drug sensitivity and resistance.

### Supplementary information

Supplementary Table 1

Supplementary Table 2

Supplementary Table 3

## Data Availability

Computational tools to process data and plot figures shown in the paper are available on https://github.com/labsyspharm/lincs_proteomics_data_descriptor and https://github.com/datarail/msda.
