# Supplementary material for: Proteomic profiling across breast cancer cell lines and models
Source: Sci Data. 2023 Aug 4;10:514. doi: 10.1038/s41597-023-02355-0 (PMC10403526; doi:10.1038/s41597-023-02355-0)
Supplement: Supplementary file 1 — Supplementary Table 1 [file 41597_2023_2355_MOESM1_ESM.docx]

**Supplementary Table 1:** Growth media and conditions for all cell lines used

| *Cell line* | *Growth media* | *Growth conditions* |
| --- | --- | --- |
| 184A1 | MEBM (CC-3150) + 1% FBS + 1% P/S | 37˚C, 5% CO_2_ |
| 184B5 | MEBM (CC-3150) + 1 ng/ml CT + 1% P/S | 37˚C, 5% CO_2_ |
| AU-565 | RPMI-1640 + 10% FBS + 1% P/S | 37˚C, 5% CO_2_ |
| BT-20 | EMEM + 10% FBS + 1% P/S | 37˚C, 5% CO_2_ |
| BT-474 | RPMI-1640 + 10% FBS + 1% P/S | 37˚C, 5% CO_2_ |
| BT-483 | RPMI-1640 + 20% FBS + 1% P/S, 0.01 mg/ml BI | 37˚C, 5% CO_2_ |
| BT-549 | RPMI-1640 + 10% FBS + 1% P/S, 1 ug/ml IN | 37˚C, 5% CO_2_ |
| CAL-120 | DMEM + 10% FBS + 1% P/S | 37˚C, 5% CO_2_ |
| CAL-51 | DMEM + 20% FBS + 1% P/S | 37˚C, 5% CO_2_ |
| CAL-85-1 | DMEM + 10% FBS + 1% P/S | 37˚C, 5% CO_2_ |
| CAMA-1 | EMEM + 10% FBS + 1% P/S | 37˚C, 5% CO_2_ |
| EFM-19 | RPMI-1640 + 10% FBS + 1% P/S | 37˚C, 5% CO_2_ |
| EVSA-T | EMEM + 10% FBS + 2 mM L-glutamine, 1% P/S | 37˚C, 5% CO_2_ |
| HCC1143 | RPMI-1640 + 10% FBS + 1% P/S | 37˚C, 5% CO_2_ |
| HCC1187 | RPMI-1640 + 10% FBS + 1% P/S | 37˚C, 5% CO_2_ |
| HCC1395 | RPMI-1640 + 10% FBS + 1% P/S | 37˚C, 5% CO_2_ |
| HCC1419 | RPMI-1640 + 10% FBS + 1% P/S | 37˚C, 5% CO_2_ |
| HCC1428 | RPMI-1640 + 10% FBS + 1% P/S | 37˚C, 5% CO_2_ |
| HCC1500 | RPMI-1640 + 10% FBS + 1% P/S | 37˚C, 5% CO_2_ |
| HCC1569 | RPMI-1640 + 10% FBS + 1% P/S | 37˚C, 5% CO_2_ |
| HCC1806 | RPMI-1640 + 10% FBS + 1% P/S | 37˚C, 5% CO_2_ |
| HCC1937 | RPMI-1640 + 10% FBS + 1% P/S | 37˚C, 5% CO_2_ |
| HCC1954 | RPMI-1640 + 10% FBS + 1% P/S | 37˚C, 5% CO_2_ |
| HCC202 | RPMI-1640 + 10% FBS + 1% P/S | 37˚C, 5% CO_2_ |
| HCC38 | RPMI-1640 + 10% FBS + 1% P/S | 37˚C, 5% CO_2_ |
| HCC70 | RPMI-1640 + 10% FBS + 1% P/S | 37˚C, 5% CO_2_ |
| hTERT-HME1 | MEMB + Lonza CC-3150 kit | 37˚C, 5% CO_2_ |
| Hs 578T | DMEM + 10% FBS + 1% P/S | 37˚C, 5% CO_2_ |
| MCF 10A | DMEM/F12 (1:1) + 5% HS + 1% P/S, 20 ng/ml EGF, 0.5 mg/ml HC, 10 µg/ml IN, 100 ng/ml CT | 37˚C, 5% CO_2_ |
| MCF12A | DMEM/F12 + 5% HS + 1% P/S, 20 ng/ml EGF, 0.5 mg/ml HC, 10 µg/ml IN, 100 ng/ml CT | 37˚C, 5% CO_2_ |
| MCF7 | DMEM + 10% FBS + 1% P/S | 37˚C, 5% CO_2_ |
| MDA-MB-134-VI | L-15 + 20% FBS + 1% P/S | 37˚C, 0% CO_2_ |
| MDA-MB-157 | L-15 + 10% FBS + 1% P/S | 37˚C, 0% CO_2_ |
| MDA-MB-175-VII | L-15 + 10% FBS + 2mM L-glutamine, 1% P/S | 37˚C, 0% CO_2_ |
| MDA-MB-231 | DMEM + 10% FBS + 1% P/S | 37˚C, 5% CO_2_ |
| MDA-MB-330 | L-15 + 20% FBS + 2mM L-glutamine,1% P/S + 30 ng/ ml EGF + 0.016 mg/ml IN + 2mM glutathione | 37˚C, 0% CO_2_ |
| MDA-MB-361 | L-15 + 20% FBS + 1% P/S | 37˚C, 0% CO_2_ |
| MDA-MB-415 | L-15 + 15% FBS + 2mM L-glutamine,1% P/S, 10 µg/ml IN | 37˚C, 0% CO_2_ |
| MDA-MB-436 | L-15 + 10% FBS + 1% P/S, 10 µg/ml IN | 37˚C, 0% CO_2_ |
| MDA-MB-453 | L-15 + 10% FBS + 1% P/S | 37˚C, 0% CO_2_ |
| MDA-MB-468 | L-15 + 10% FBS + 1% P/S | 37˚C, 0% CO_2_ |
| MGH312 | RPMI + 10% FBS + 1% P/S | 37˚C, 5% CO_2_ |
| MGH358 | RPMI + 10% FBS + 1% P/S | 37˚C, 5% CO_2_ |
| PDX1206 | DMEM/F12 (3:1) + 7.5% FBS + 1% P/S, 0.125 ng/ml EGF, 25 ng/ml HC, 5 µg/ml IN, 8.6 ng/ml CT, 5 uM Y-27632 | 37˚C, 5% CO_2_ |
| PDX1258 | DMEM/F12 (3:1) + 7.5% FBS + 1% P/S, 0.125 ng/ml EGF, 25 ng/ml HC, 5 µg/ml IN, 8.6 ng/ml CT, 5 uM Y-27632 | 37˚C, 5% CO_2_ |
| PDX1328 | DMEM/F12 (3:1) + 7.5% FBS + 1% P/S, 0.125 ng/ml EGF, 25 ng/ml HC, 5 µg/ml IN, 8.6 ng/ml CT, 5 uM Y-27632 | 37˚C, 5% CO_2_ |
| PDXHCI002 | DMEM/F12 (3:1) + 7.5% FBS + 1% P/S, 0.125 ng/ml EGF, 25 ng/ml HC, 5 µg/ml IN, 8.6 ng/ml CT, 5 uM Y-27632 | 37˚C, 5% CO_2_ |
| SK-BR-3 | McCoy's + 10% FBS + 1% P/S | 37˚C, 5% CO_2_ |
| SUM1315MO2 | F-12 + 5% FBS + 1% P/S, 10 ng/ml EGF, 5 µg/ml IN, 10 mM HEPES | 37˚C, 5% CO_2_ |
| SUM149PT | F-12 + 5% FBS + 1% P/S, 1 µg/ml HC, 5 µg/ml IN, 10 mM HEPES | 37˚C, 5% CO_2_ |
| SUM159PT | F-12 + 5% FBS + 1% P/S, 1 µg/ml HC, 5 µg/ml IN, 10 mM HEPES | 37˚C, 5% CO_2_ |
| SUM-185PE | Ham's F12 + 5% FBS + 1% P/S + 5 µg/ml BI + 1 µg/ml HC + 10 mM HEPES | 37˚C, 5% CO_2_ |
| SUM-190PT | Ham's F12 + 1% FBS + 1% P/S, 5 µg/ml BI, 1 µg/ml HC, 10 mM Hepes, 5 mM ethanolamine, 5 µg/ml transferrin, 10 nM triiodo thyronine, 50 nM sodium selenite, 1 g/l BSA | 37˚C, 5% CO_2_ |
| SUM-44PE | Ham's F12 + 1% FBS + 1% P/S, 5 µg/ml BI, 1 µg/ml HC, 10 mM HEPES, 5 mM ethanolamine, 5 µg/ml transferrin, 10 nM triiodo thyronine, 50 nM sodium selenite, 1 g/l BSA | 37˚C, 5% CO_2_ |
| SUM-52PE | Ham's F12 + 5% FBS + 1% P/S + 5 µg/ml BI + 1 µg/ml HC + 10 mM HEPES | 37˚C, 5% CO_2_ |
| T47D | RPMI-1640 + 10% FBS + 1% P/S, 1 µg/ml IN | 37˚C, 5% CO_2_ |
| UACC-812 | L-15 + 20% FBS + 2mM L-glutamine, 1% P/S, 20 ng/ml EGF | 37˚C, 0% CO_2_ |
| UACC-893 | L-15 + 10% FBS + 1% P/S | 37˚C, 0% CO_2_ |
| ZR-75-1 | RPMI-1640 + 10% FBS + 1% P/S | 37˚C, 5% CO_2_ |
| ZR-75-30 | RPMI-1640 + 10% FBS + 1% P/S | 37˚C, 5% CO_2_ |

Abbreviations: fetal bovine serum (FBS), penicillin/streptomycin (P/S), insulin (IN), bovine insulin (BI), hydrocortisone (HC), epidermal growth factor (EGF), cholera toxin (CT).
